# Fine mapping of candidate quantitative trait loci for plant and ear height in a maize nested-association mapping population

**DOI:** 10.3389/fpls.2022.963985

**Published:** 2022-08-04

**Authors:** Xingfu Yin, Yaqi Bi, Fuyan Jiang, Ruijia Guo, Yudong Zhang, Jun Fan, Manjit S. Kang, Xingming Fan

**Affiliations:** ^1^College of Agronomy and Biotechnology, Yunnan Agricultural University, Kunming, China; ^2^Institute of Food Crops, Yunnan Academy of Agricultural Sciences, Kunming, China; ^3^Department of Plant Pathology, Kansas State University, Manhattan, KS, United States

**Keywords:** plant height, ear height, maize, nested-association mapping, genome-wide association study

## Abstract

Plant height (PH) and ear height (EH) are two important traits in maize (*Zea mays* L.), as they are closely related to lodging resistance and planting density. Our objectives were to (1) investigate single-nucleotide polymorphisms (SNPs) that are associated with PH and EH for detecting quantitative trait loci (QTL) and new gene that determines PH and EH, (2) explore the value of the QTL in maize breeding, and (3) investigate whether the “triangle heterotic group” theory is applicable for lowering PH and EH to increase yield. Seven inbred female parents were crossed with a common founder male parent Ye 107 to create a nested association mapping (NAM) population. The analysis of phenotypic data on PH and EH revealed wide variation among the parents of the NAM population. Genome-wide association study (GWAS) and high-resolution linkage mapping were conducted using the NAM population, which generated 264,694 SNPs by genotyping-by-sequencing. A total of 105 SNPs and 22 QTL were identified by GWAS and found to be significantly associated with PH and EH. A high-confidence QTL for PH, *Qtl-chr1-EP*, was identified on chromosome 1 *via* GWAS and confirmed by linkage analysis in two recombinant inbred line (RIL) populations. Results revealed that the SNP variation in the promoter region of the candidate gene *Zm00001d031938*, located at *Qtl-chr1-EP*, which encoded UDP-N-acetylglucosamine-peptide N-acetyl-glucosaminyl-transferase, might decrease PH and EH. Furthermore, the triangle heterotic pattern adopted in maize breeding programs by our team is practicable in selecting high-yield crosses based on the low ratio of EH/PH (EP).

## Introduction

Maize (*Zea mays* L.) is one of the most important crops in the world; it is used for food, feed, fuel, as well as raw material for maize-based industry (Duvick, 2005; Li et al., 2020). Besides, it is also a vital model plant species that is endowed with a high level of phenotypic and genetic diversity. Many agronomic traits in maize have been improved during 1000 of years of domestication and selection. Because of the target-specific domestication under specific environments across the world, an unintended consequence was the loss of genome-wide diversity at unselected loci (Wang et al., 2005). Studies on gene sequences and isozymes confirmed the reduction in genetic variation in maize (Doebley et al., 1984; Eyre-Walker et al., 1998). The use of molecular markers with proper populations has helped scientists to better understand the genetic effects on target traits (Gore et al., 2009; Chia et al., 2012).

To increase productivity, the diverse genetic background of parents is required to prevent the loss of important genes in maize. Many genes have been shown to be involved in the modification of the aboveground architecture of maize (Voorend et al., 2016; Xiao et al., 2021). Since the 1950s, scientists have made a great effort for reducing the plant height (PH) of maize to substantially increase the plant density, which, in turn, increases grain yield (Teng et al., 2013; Bishopp and Lynch, 2015; Abdel-Ghani et al., 2016). Plant height and ear height (EH) are two important traits that directly influence plant architecture, and have been manipulated during maize domestication since they are significantly correlated with grain yield (Teng et al., 2013; Abdel-Ghani et al., 2016). Modern molecular technologies for studying PH and EH provide maize breeders new insights into their breeding programs for developing high-yielding varieties.

Genome-wide association study is a tool for characterizing quantitative trait loci (QTL). The tool has shown an enormous potential for exploring QTL with high resolution in various germplasm (Buntjer et al., 2005). However, the results from GWAS might be confounded with spurious associations (false positives) possibly caused by population stratification and cryptic relatedness (Platt et al., 2010; Do et al., 2019; Sesia et al., 2021). The linkage mapping of QTL is another tool based on bi-parental populations and it has also been used in maize for genetic dissection of complex traits (Blanc et al., 2008; Stange et al., 2013; Lian et al., 2014), including PH and EH in maize (Li et al., 2016). With the enhancements in sequencing technologies, many researches have indicated that a multi-parental population is highly desirable for detecting QTL. Multi-parental mapping populations or the next-generation mapping populations, such as nested-association mapping (NAM), have already shown their huge potential in maize (Yu et al., 2008; Gage et al., 2020), wheat (Mackay et al., 2014), and soybean (Beche et al., 2020). The NAM methodology was initially developed to enable dissection of the genetic architecture of complex traits with high confidence and high resolution by incorporating the best features of the previous two approaches (Zhang et al., 2005; McMullen et al., 2009). It also allows validation of mapping results through the subpopulations within a NAM population. By utilizing the maize NAM population, QTL of many traits, such as flowering time, male and female inflorescence traits, leaf architecture, southern corn leaf blight resistance, and northern corn leaf blight resistance, have been identified (Buckler et al., 2009; Brown et al., 2011; Kump et al., 2011; Tian et al., 2011; Poland et al., 2012). Availability of a high-density genotyping platform with uniformly distributed genome-wide genetic markers was useful for high-resolution genetic dissection of complex traits and tracking of favorable alleles in many populations (Pandey et al., 2012; Varshney et al., 2013). Combination of GWAS and linkage mapping provided enhanced mapping resolution and reduced the time and cost of developing synthetic mapping populations (Flint-Garcia et al., 2005; Wu et al., 2020), which have been applied to QTL identification.

The use of a multi-parent population, such as NAM, could not only detect QTL but also individual functional genes. These identified QTL and candidate genes can then serve as useful markers for future breeding programs and facilitate breeders to further understand the regulatory mechanisms of PH and EH in maize. Peiffer et al. (2014) had used a NAM population and representative inbreds in the United States to study PH and EH in maize. They found that PH was under a strong genetic control and had a highly polygenic genetic architecture. Several significant associations between QTL and PH loci were identified, such as brassinosteroid-deficient *dwarf1*, *dwarf plant1*, and *semi-dwarf2* (Best et al., 2016; Castorina et al., 2018; Wei et al., 2018).

To better understand the inheritance of PH and EH and to apply the results to maize breeding programs, we developed a NAM population by crossing seven diverse female parents (TRL02, CML373, CML312, CML395, Q11, D39, and Y32) with a common founder Ye107 (as a male parent). The objectives of this study were to (1) investigate SNPs associated with PH and EH for detecting QTL and new gene that condition PH and EH, (2) explore the application of QTL to maize breeding programs, and (3) investigate whether the “triangle heterotic group” theory is applicable to PH and EH in maize breeding programs.

## Materials and methods

### Population development

**Figure 1:**
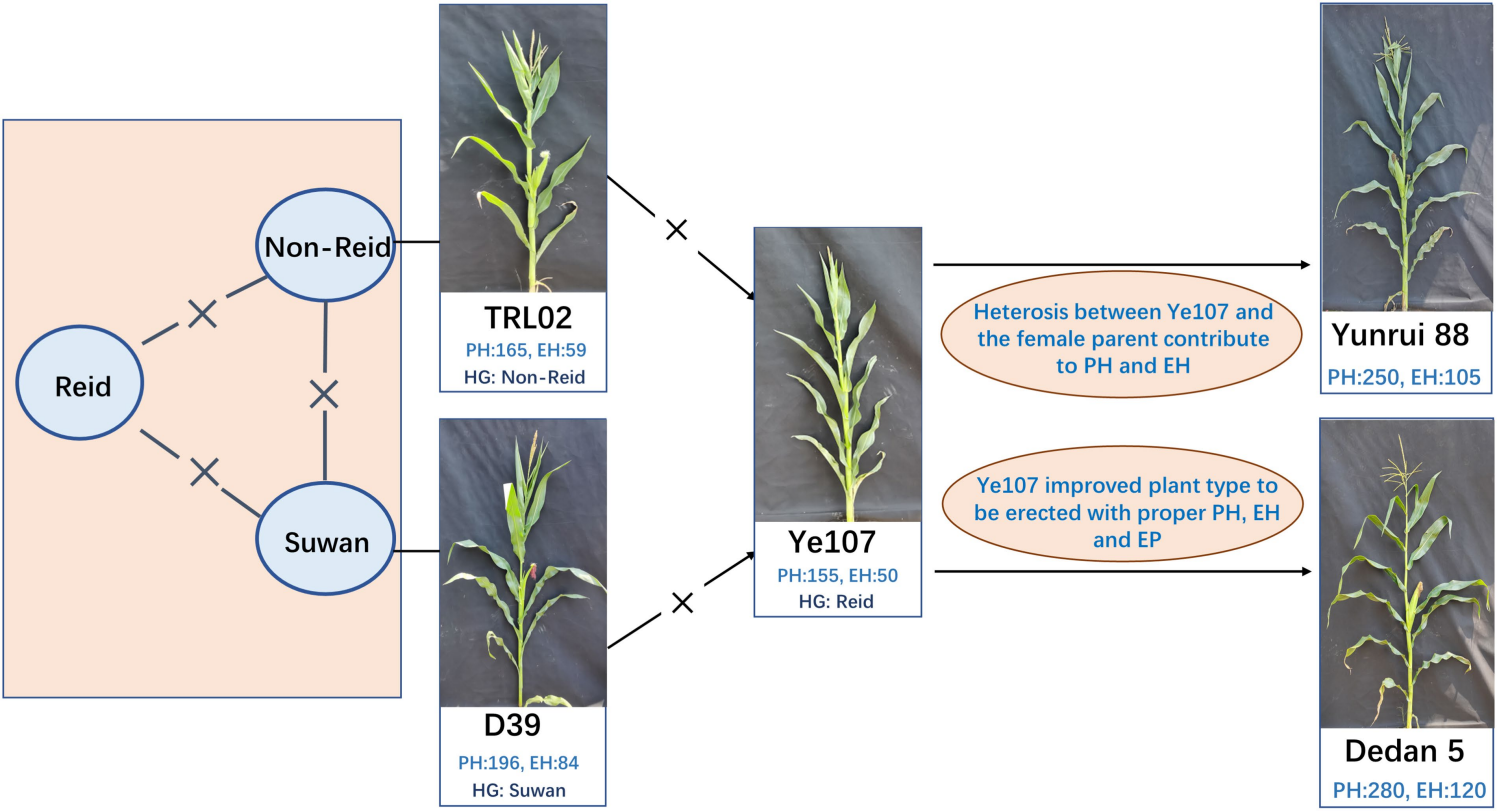
Breeding strategy of the two hybrids by applying triangle heterotic pattern using TRL02, D39, and Ye107 (Fan et al., 2014).

The maize NAM population was derived from a cross between founder parent Ye107 and seven diverse parents. The common founder (Ye107) is a key elite inbred lines developed with two lines from different heterotic groups (Figure 1) in Chinese breeding programs. Ye107 has served as a parent in many commercial hybrids that have been planted in a quite large production area in China (Zhen et al., 2004). In a previous study, authors had proposed the “triangle heterotic pattern” as a breeding strategy to develop maize hybrids with improved efficiency (Fan et al., 2014). Ye107 is a temperate maize inbred line belonging to the Reid heterotic group, with erect plant type, relatively low PH and EH, tolerance to high density, and high combining ability for grain yield. In this NAM study, we used Ye107 as a common parent in crosses with seven tropical and subtropical lines that belonged to Suwan1 or Non-Reid heterotic groups. The pedigree and heterotic groups of the parental lines are listed in Table 1. Seven RILs (*F*_2:7_) were generated and a sample of ~200 lines was randomly selected from each RIL to construct a NAM population. During the selfing process, some of the inbred lines were lost due to inbreeding depression and other stresses. Eventually, 1,215 RILs were used for this study.

**Table 1:** Information on the common parent (Ye107) and seven lines that were crossed to it for developing seven nested-association mapping populations.

NAM	Parents	Pedigree	Heterotic group	Ecological type
	Ye107	Selected from US hybrid DeKalb XL80	Reid	Temperate
NAM-1	Q11	Derived from US hybrid	Reid	Subtropical
NAM-2	TRL02	Derived from US hybrid	Non-Reid	Subtropical
NAM-3	CML373	P43SR-4-1-1-2-1-B-8-1-B	Non-Reid	Tropical
NAM-4	CML312	S89500-F2-2-2-1-1-B	Non-Reid	Tropical
NAM-5	CML395	90323B-1-B-1-B	Non-Reid	Tropical
NAM-6	D39	Selected from Suwan11	Suwan1	Tropical
NAM-7	Y32	Selected from Suwan11	Suwan1	Tropical

### Field experiment and phenotypic data analysis

The population was planted at Dehong (DH) and Baoshan (BS) in Yunnan Province, China, in 2019. A randomized complete block design with two replications was employed at each location. Each experimental plot consisted of two 3-m-long rows, with an inter-row spacing of 0.70 m and 14 plants per row. The overall plant density was ~62,112 plants ha^−1^. Trials were managed according to standard local practices. Ten plants from the middle of each row were sampled. Data on PH and EH were subjected to best linear unbiased prediction (BLUP) using the lme4 package in R (v3.2.2).

### Heterosis analysis

The heterosis of each RIL population was calculated as follows:


MPH=[(F1−MP)/MP]×100

Where MPH is the mid-parent heterosis, *F*_1_ is the target trait value of hybrid, and MP is the target trait value of the mean of parents (Ali et al., 2012).

### Genotyping by sequencing

Genomic DNA was extracted from seedling leaves of each accession using an improved cetyl trimethyl ammonium bromide method with slight modifications: 4 mM tris (2-carboxyethyl) phosphine was used in place of 2-mercaptoethanol and supplemented with 2% polyvinylpolypyrrolidone and 40 mg RNase (Stewart and Via, 1993). DNA concentration was quantified using the Quant-iT PicoGreen dsDNA Assay Kit (Life Technologies, Grand Island, NY, United States) and normalized to 20 ng/ml for library construction.

The GBS libraries were constructed according to Poland et al. (2012). In brief, genomic DNA was digested using the restriction enzymes PstI and MspI (New England BioLabs, Ipswich, MA, United States), and barcoded adapters were ligated to the digested DNA fragments using T4 ligase (New England BioLabs). All ligated products from each plate were pooled and cleaned using the QIAquick PCR Purification Kit (QIAGEN, Valencia, CA, United States). Primers complementary to both adaptors were used for PCR. The PCR products were then cleaned with the QIAquick PCR Purification Kit and quantified using the Qubit dsDNA HS Assay Kit (Life Technologies). After size selection for 200-bp to 300-bp fragments in an Egel system (Life Technologies), the concentration of each library was estimated with a Qubit 2.0 fluorometer and the Qubit dsDNA HS Assay Kit (Life Technologies). The size-selected library was sequenced on an Ion Proton sequencer (Life Technologies, software version 5.10.1) by using P1v3 chips after library preparation on an Ion Chef instrument (Ion PI HiQ Chef Kit). The Ion Torrent system produces sequence reads of variable lengths. Before TASSEL 5.0 analysis, 80 poly (A) bases were appended to the 30 ends of all sequencing reads so that TASSEL 5.0 would attempt to use the reads shorter than 64 bases rather than discard short reads. Single-nucleotide polymorphisms were then called with the Genome Analysis Toolkit software (McKenna et al., 2010) and the B73 reference genome (Jiao et al., 2017). B73 is the most important source of the reference maize genome sequence (Schnable et al., 2009). The identified SNPs were further annotated with the ANNOVAR software tool (v2013-05-20; Wang et al., 2010).

### Genome-wide association study

Genome-wide association study was used to map QTL in the NAM population. The GEMMA (genome-wide efficient mixed-model association) software package and its mixed-linear-model analysis method were applied for the GWAS analysis (Zhou and Stephens, 2012) with a significance threshold of *p* < 0.00001. The genome-wide average block length was 18,825 bp obtained by using plink software (v1.9)^1^ with the following parameters: –blocks no-pheno-req –blocks-max-kb 200 –blocks-min-maf 0.05 –blocks-strong-lowci 0.70 –blocks-strong-highci 0.98 –blocks-recomb-highci 0.90 –blocks-inform-frac 0.95 to identify SNPs associated with maize PH and EH. Plant height-and EH-related candidate genes located within 20 Kb around the peak SNP were determined on the basis of examination of the B73 reference genome. Maize GDB and NCBI were used for annotation and functional predictive analyses of the candidate genes.

### Linkage mapping and QTL analysis

The RIL populations of Ye107 with CML312 (with lowest EP in all populations), Y32 (with highest EP in all populations), and D39 (with medium EP in all populations) were genotyped by GBS-based SNPs. Allelic SNPs with different genotypes in parents were then used to construct paternal and maternal linkage maps with the JoinMap 4 software. An LOD score cutoff of 5.0 was used to determine linkage groups. Composite interval mapping (CIM) for QTL mapping was performed *via* Windows QTL Cartographer 2.0 (Zeng, 1994). The threshold value was set with 1,000 random permutations (Churchill and Doerge, 1994). The determination of the QTL region that contained the marker with LOD > 2.5 related to its flanking markers. The proportion of phenotypic variation explained by a single QTL was determined by the square of the partial correlation coefficient (*R*^2^).

## Results

### Phenotypic evaluation and heterosis for PH and EH

**Figure 2:**
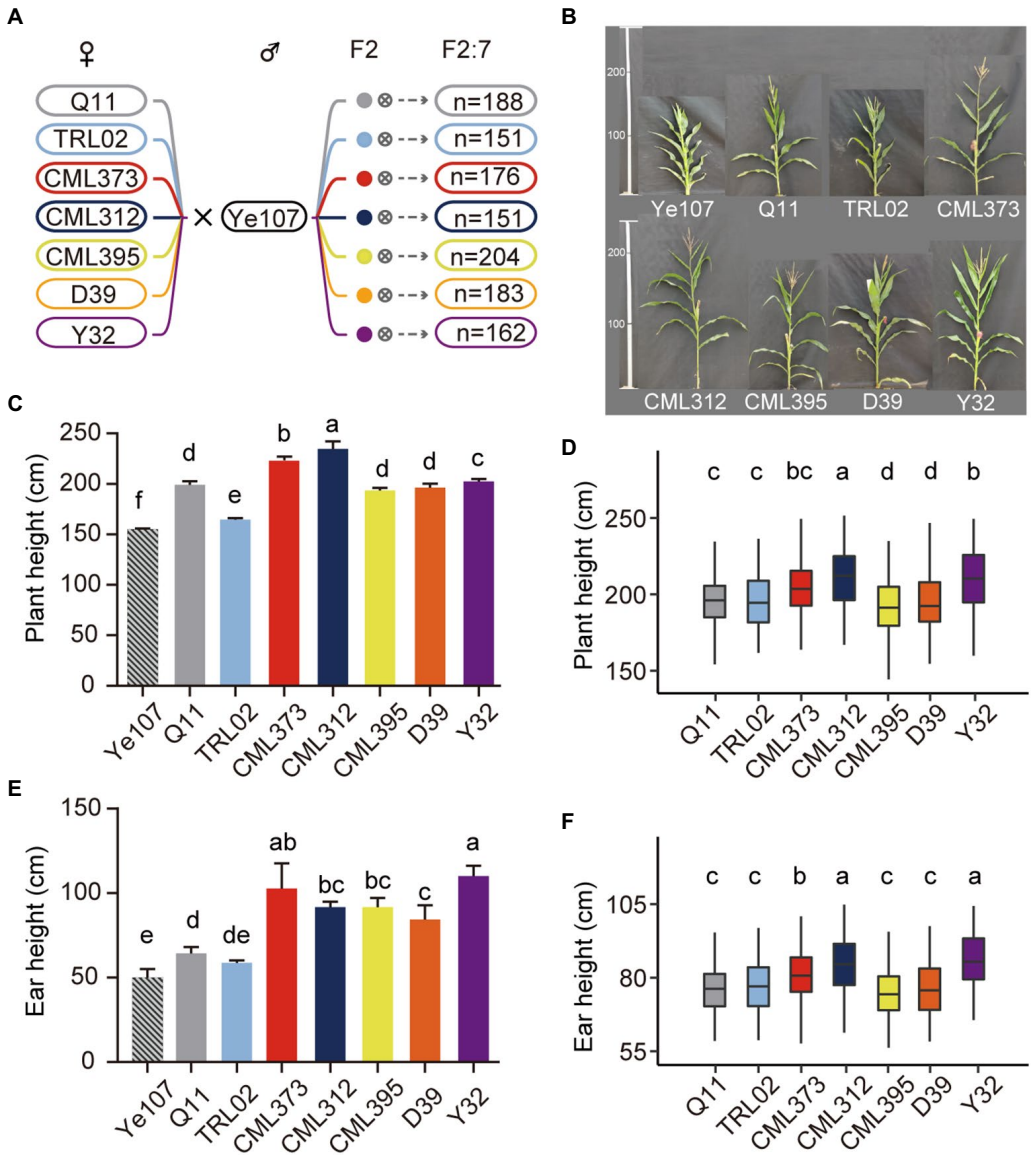
The population construction and morphological comparison of the plant height. **(A)** The number of families for each of the RIL population. **(B)** Phenotypes of each parental line. **(C)** Plant height of each parental line. **(D)** Plant height of each recombinant inbred line population. **(E)** Ear height of each parental line. **(F)** Ear height of each recombinant inbred line population.

A NAM population of 1,215 maize RILs, consisting of seven *F*_2:7_ RILs, is shown in Figure 2A. The number of RILs in each cross ranged from 151 in the TRL02 × Ye107 RILs to 204 in the CML395 × Ye107 RILs (Figure 2A). Phenotypic measurements were carried out for PH and EH at Dehong and Baoshan in Yunnan Province, China, in 2019.

A high level of phenotypic variation was observed among parents and RILs (Figure 2B). Significant genotypic differences were detected for PH between male parent Ye107 and each female parent (Figure 2C). The PH of all female parents (ranging from 164.67 cm to 228.33 cm) was significantly higher than that of the male parent Ye107 (155.00 cm; Figure 2C). Data showed that there were extensive phenotypic variations for PH and EH among the RIL populations. The offspring from CML312 family had the highest PH (mean = 176.51 cm in the DH environment and 172.99 cm in the BS environment), and the TRL02 had the lowest PH (mean = 156.64 cm in DH and 141.92 cm in BS). With data from BLUP, offspring from CML312 and CML395 families had the highest and lowest PH (Figure 2D). Similar to PH, EH also had large phenotypic variation among parents (Figure 2E). All female lines had higher EH than that of the male parent. In addition, there were significant differences among female parents (Figure 2E). Interestingly, though the PH of TRL02 was the lowest among all female parents (Figure 2E), the mean PH and mean EH of its offspring RIL population (Figure 2F) were higher than those of the offspring from other parents except the mean PH for CML312 family. These results indicated that the PH of offspring was not only affected by the height of their parents, but also by the very combination of the different parents. Therefore, the NAM population, composed of diverse multi-parental cross combinations, was needed and was suitable for QTL mapping because the mapping with this NAM population could eliminate the possible false-positive events caused by using only one specific cross from two parents.

**Figure 3:**
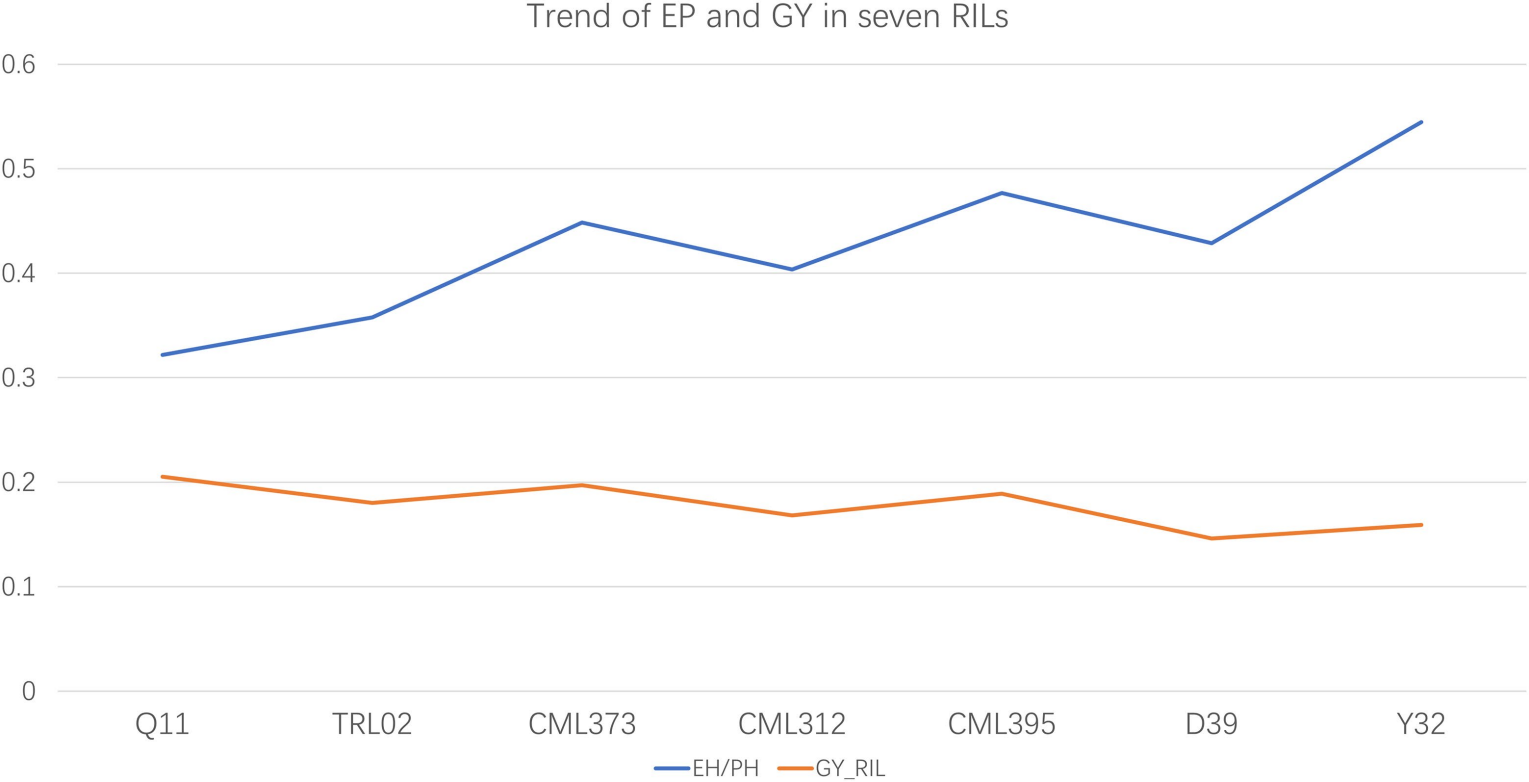
The trends of grain yield and ratio of ear height and plant height are in opposite direction in the seven recombinant inbred line populations at current plant density.

The MPH for PH and EH of the *F*_1_ hybrids is listed in Table 2. The result shows that TRL02 × Ye107 had the highest MPH for EH and the second-highest MPH for PH. In field, though TRL02 was the shortest in PH and EH among the seven female parents, the offspring was not significantly different in PH from Q11 and CML395 and in EH from Q11, CML395, and D39. Correlation analysis showed that there was no statistically significant relationship for PH and EH between parents and their RIL populations. However, if we plot the EP and GY of seven RILs (Figure 3), we find that the trends for EP and GY are in opposite directions. Since this experiment was not designed specially at multiple plant densities, the GY potential of each hybrid at different densities was not available. Thus, a solid statistical relationship could not be established between GY and EP in the RIL populations.

**Table 2:** The mid-parent heterosis of plant height, ear height and yield for each recombinant inbred line population.

Line	Plant height			Ear height			Yield of *F*_1_		
	PH	PH of *F*_1_	MPH	EH	EH of *F*_1_	MPH	Yield	Yield of *F*_1_	MPH
Q11	199	215	0.21	64	80	0.40	0.093	0.205	1.18
TRL02	165	212	0.33	59	83	0.52	0.121	0.180	1.98
CML373	223	230	0.22	100	88	0.17	0.120	0.197	2.28
CML312	228	245	0.28	92	95	0.34	0.123	0.168	1.73
CML395	193	207	0.19	92	75	0.06	0.130	0.189	1.91
D39	196	210	0.20	84	78	0.16	0.097	0.146	2.01
Y32	202	250	0.40	110	100	0.25	0.106	0.159	2.00
Ye107	155	–	–	50	–		0.095	–	

### Genomic variation in RIL populations and population structures

A total of 264,694 high-quality SNPs were discovered with GBS across seven RIL populations. The genome-wide mean SNP density was 133.15 SNPs per Mb, evenly distributed along the chromosomes. Single-nucleotide polymorphism density varied from 125.06 SNPs per Mb on chromosome 2–139.33 SNPs per Mb on chromosome 4 (Supplementary Figure 1). Based on the B73 reference genome annotation (RefGen_v4), these SNPs were mainly located in intergenic regions (86.1%), followed by introns (5.76%), gene coding sequences (2.43%), 3′ UTRs (1.19%), and 5′ UTRs (0.72%; Supplementary Table 1).

**Figure 4:**
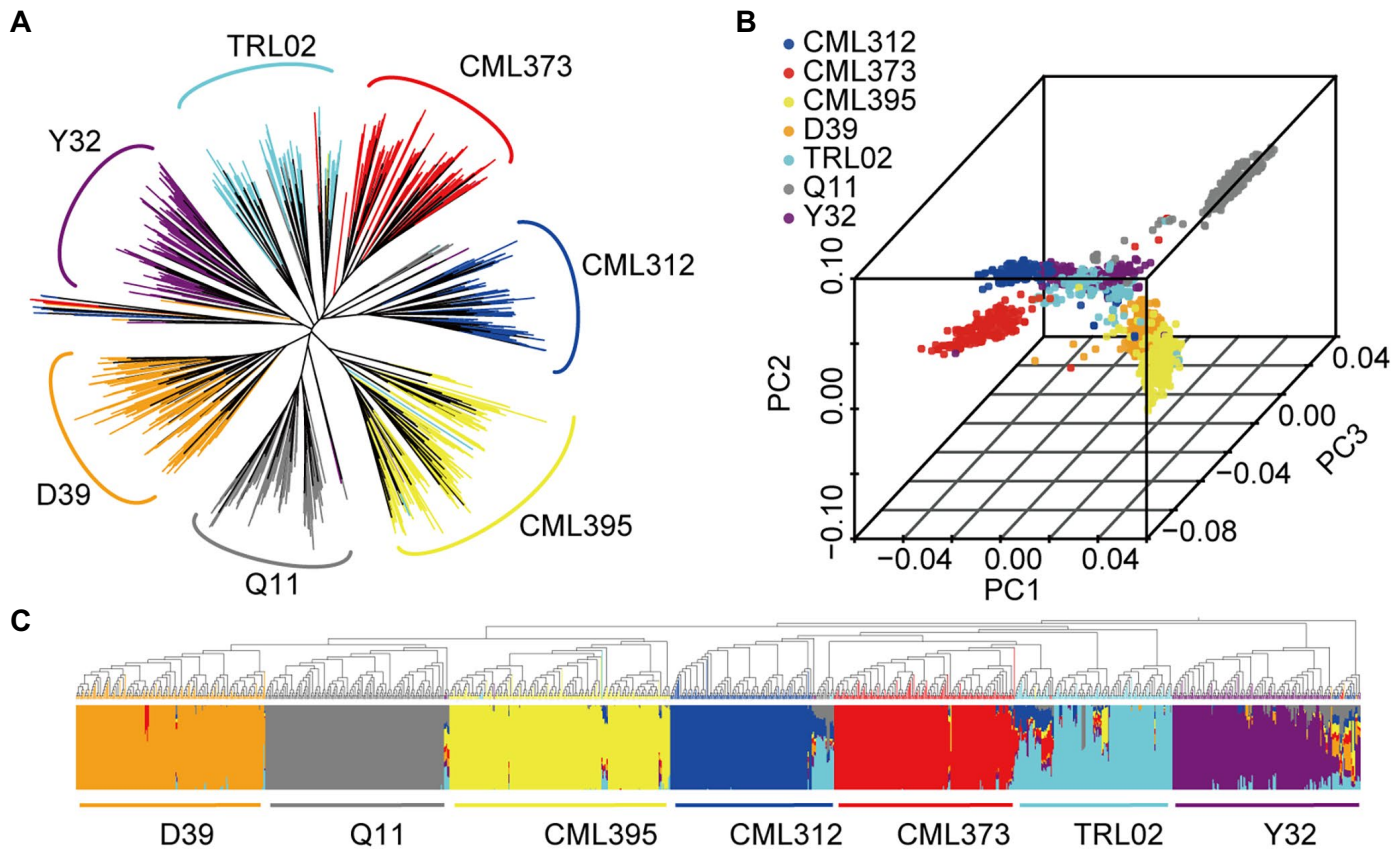
Phylogenetic tree **(A)**, principal component analysis **(B),** and genetic structure **(C)** of the 1,215 accessions.

Although the founder parents were all cultivated maize, NAM produced a particular phylogenetic relationship that was clearly classified according to the founder’s parents (Figure 4A). Accordingly, analyses of principal components and population structure revealed that the NAM population could also be divided into seven groups. However, the seven groups were divided into three clusters. This further supports our previously published results on three heterotic pattern theory (Fan et al., 2014). Here, we classified Q11 as belonging to Reid; D39, CML395, and Y32 belong to Suwan1, and the remainder three lines belong to Non-Reid heterotic group (Figures 4B,C). There are some lines mixed at the middle of the clusters (Figure 4B), which might suggest that the introgression of genes occurred during breeding (Beck et al., 1991). Different clusters or heterotic groups may have different gene frequencies; therefore, population structure, a possible factor to cause false-positive associations in GWAS (Sul et al., 2018), was taken into account in the subsequent analysis.

### Genome-wide association study analysis for PH and EH

**Figure 5:**
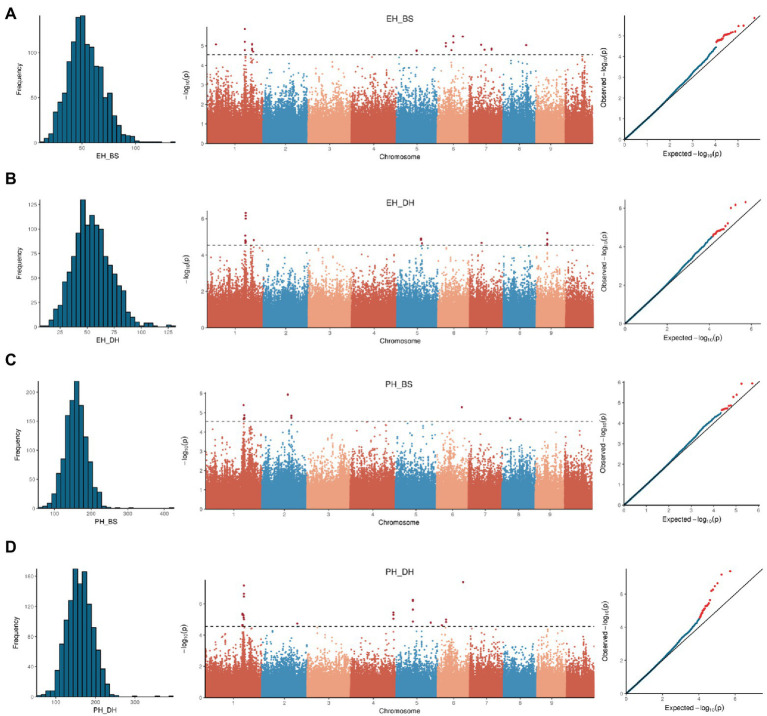
Phenotypic distribution, Manhattan and QQ-plot of genome-wide association study for ear height in Baoshan **(A)**, ear height in Dehong **(B)**, plant height in Baoshan **(C),** and plant height in Dehong **(D)**.

The 264,694 SNPs, with a missing value <0.2 and a minor allele frequency (MAF) > 0.05, were used for GWAS. Analysis results, being consistent with the clear stratification of population structure, suggested that the CV error shows a turning point on the slope at *K* = 7 (Supplementary Figure 2). Single-nucleotide polymorphism-based GWAS was performed on PH, EH, and EP (EH/PH) traits with MLM by incorporating both the population structure (first seven principal components, PCA) and kinship into the model. A total of 125 SNP-trait associations were detected with −log_10_(P) > 4.5, including 56 for PH, 49 for EH, and 20 for EP (Figure 5; Supplementary Figure 3; Supplementary Tables 2, 3).

Since the average block length of the whole genome is about 20 kb, QTL interval was defined as 40 kb regions surrounding GWAS signal peaks. The most significant peak (containing multiple significant SNPs) was located on chromosome 1 (207–210 Mb). The peak was significantly associated with both PH and EH in both Dehong and Baoshan. The most significant SNP for both PH and EH was chr1_210043412, with a *p*-value < 10e-6 (Figure 5).

### Mapping QTL for PH, EH, and EP

To identify QTL for PH and EH, we screened 13,936, 14,450, and 13,683 SNPs with different alleles in parents for linkage map construction in the CML312, D39, and Y32 RIL family, respectively. The markers with integrity (0.3) and segregation distortion (*p* < 0.001) were filtered. Three high-density genetic maps were constructed (Supplementary Figure 4), and these linkage maps spanned a total genetic distance of 3473.92 cM, 3017.52 cM, and 3315.86 cM in CML312, D39, and Y32 RIL family, respectively.

**Figure 6:**
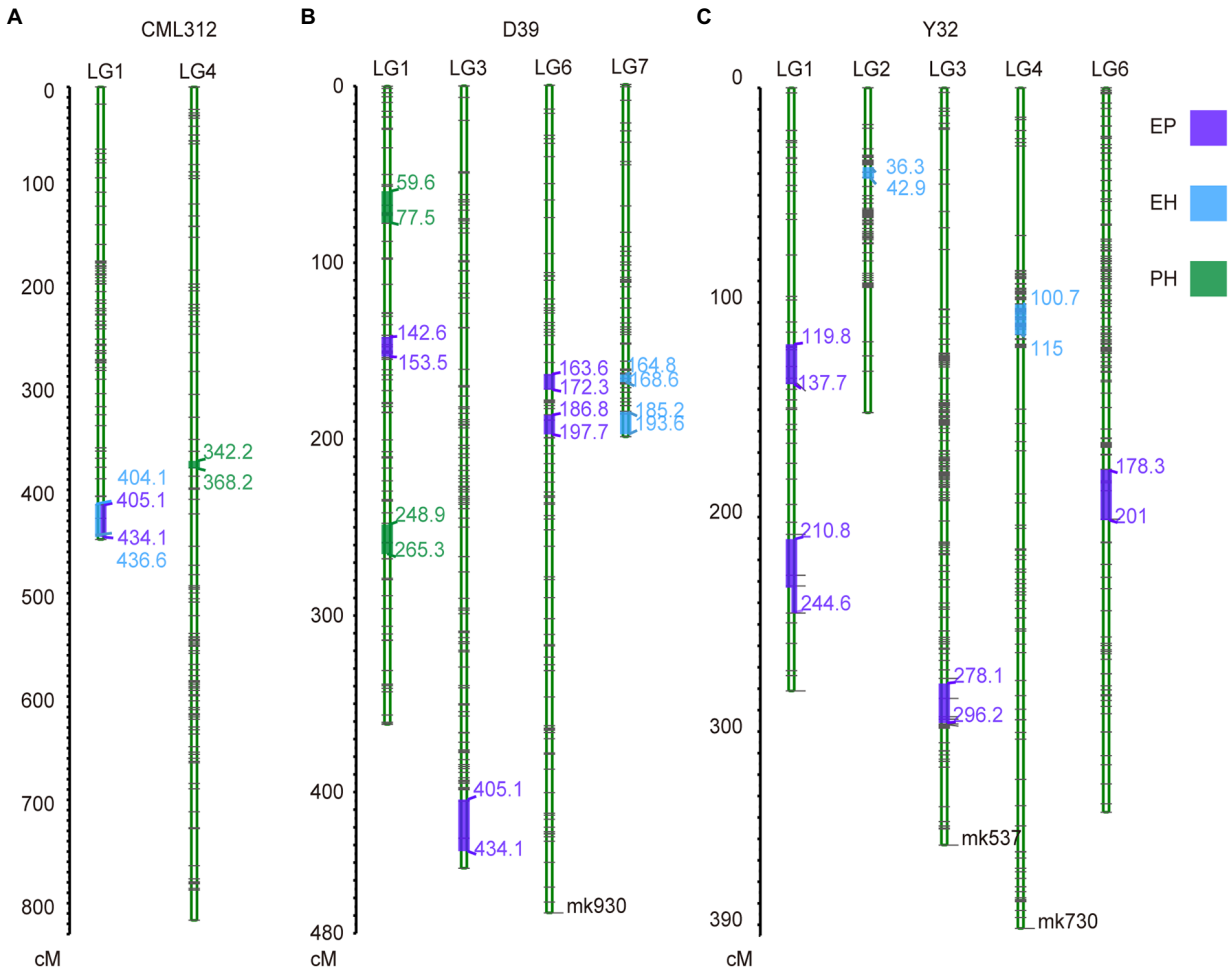
Genetic linkage map and QTL mapping for CML312 population **(A)**, D39 population **(B)** and Y32 population **(C)**.

Quantitative trait loci mapping was conducted for PH, EH, and EP using individual linkage maps from CML312, D39, and Y32 RIL families. Twenty-two QTL for PH, EH, and EP were detected (Supplementary Figures 4–6; Supplementary Table 4). For PH, one significant QTL was identified in CML312 RIL family and three in the D39 RIL family (Figures 6A,B; Supplementary Figures 5, 6), which together explained 30.7% of the observed phenotypic variance (Supplementary Table 4). For EH, one QTL was detected in CML312, two in the D39 linkage map, and three QTL in Y32 (Figures 6A–C; Supplementary Figures 5–7), which accounted for 64.92% of the phenotypic variance (Supplementary Table 4). For EP, we found nine QTL that were distributed on chromosomes 1, 3, and 6. One QTL was found in CML312, four in D39, and four in the Y32 linkage map (Figures 6A–C), accounting for 75.43% of the phenotypic variance (Supplementary Table 4).

### Identification of genes related to PH and EH

**Figure 7:**
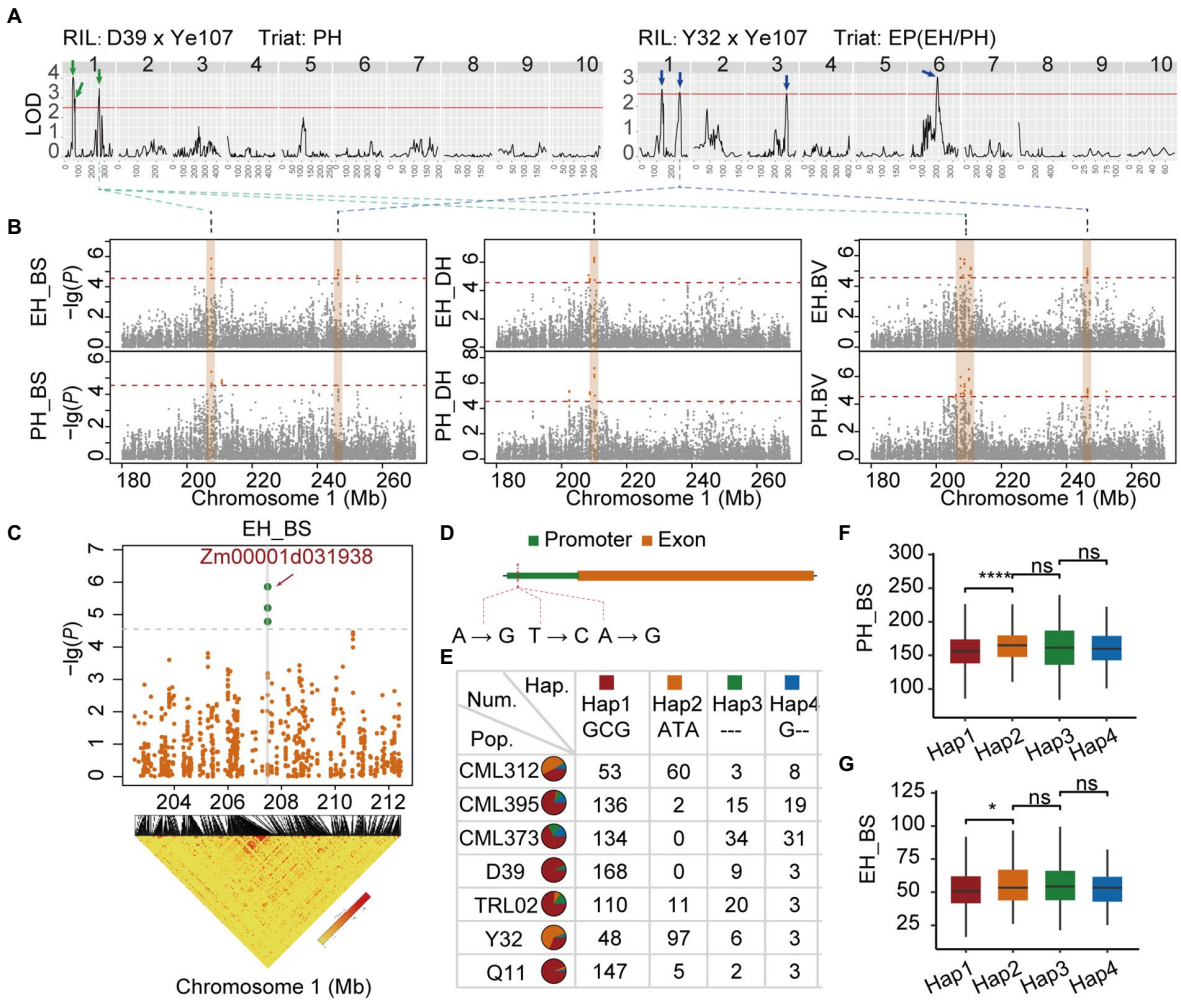
**(A)** QTL mapping of PH in D39 × Ye107 and EP in Y32 × Ye107. **(B)** Locus in Manhattan by GWAS. **(C)** Linkage disequilibrium block of *Qtl-chr1-EP*. **(D)** The three SNPs in the loci and the distribution ratio of the four haplotypes in seven RILs **(E)**. The PH **(F)** and EH **(G)** of the four haplotypes in Baoshan.

To explore the best candidate genes from the QTL identified above for PH, EH, and EP, a large QTL *Qtl-chr1-EP* located on chromosome 1 (200–260 Mb) was identified *via* QTL mapping in D39 RIL family for PH, and also in Y32 RIL family for EP (Figure 7A). We focus our investigation on the most significant GWAS signal region (207–210 Mb) of *Qtl-chr1-EP* (Figures 7A,B). *Qtl-chr1-EP* was detected for PH and EH in both Dehong and Baoshan environments *via* GWAS, also by using the BLUP for PH and EH (Figure 6B). A QTL signal of EP, being close to *Qtl-chr1-EP*, was also detected by both QTL mapping and GWAS in the Y32 RIL family (Figures 7A,B).

According to the annotation of gene information, we found that three most significant GWAS SNPs (SNP_207482464, SNP_207482494, SNP_207482507) fell into the promoter region of *Zm00001d031938* that codes probably UDP-N-acetyl-glucosamine-peptide N-acetyl-glucosaminyl transferase (Figures 7C,D). According to the genotyping results, four haplotypes [GCG, ATA, G--, and --- (all missing)] consisting of three significant SNPs, were detected in the population (Figure 7E). The proportion of Hap1 in seven RIL families ranged from 31.17% to 93.63% (Figure 7E). Haplotype analysis revealed that Hap 1 had a significant effect on reducing PH and EH (Figures 7F,G).

## Discussion

### Candidate gene for plant height in maize

A high confidence PH QTL, *Qtl-chr1-EP*, was identified with *p* value of 6.32 in RIL populations (Supplementary Table 2) from this study. It explained 10.0% of the phenotypic variation in D39 population (Supplementary Table 4).

Has this gene been involved in any important bio-processes for determining PH and EH? Xiao et al. (2021) found an epistatic interaction between QTL *Brachytic2* (*BR2*) and *ubiquitin3* (*ubi3*) contributed to the heterotic performance of PH by detecting one PH peak (peak SNP: chr1.s_201665854) located close to the region of the gene detected in our study. Candidate gene analysis showed that *Qtl-chr1-EP* was located in the promoter region of a candidate gene, *Zm00001d031938*, which encodes UDP-N-acetylglucosamine-peptide N-acetylglucosaminyl-transferase (OGT). OGTs are involved in various processes, such as gibberellin (GA) signaling pathway. OGTs catalyze the addition of nucleotide-activated sugars into the polypeptide by O-glycosidic linkage with the hydroxyl of serine or threonine (Hart et al., 2007). The Arabidopsis genome encodes two putative OGTs: SPINDLY (SPY) and SECRET AGENT (SEC). The activity of SEC had been illustrated (Kim et al., 2011). Zentella et al. (2016) showed that SEC interacted with O-GlcNAcylates DELLAs, which was a key negative regulator of GA signal determining PH. The GA pathway is a key metabolic pathway known to regulate plant height in maize. Studies showed that *Dwarf3* (*D3*), *ZmGA3ox2*, and *Anther ear1* (*An1*) were involved in the early stages of GA biosynthesis (Bensen et al., 1995; Winkler and Helentjaris, 1995; Teng et al., 2013), while *dwarf8* and *dwarf9* regulate DELLA proteins of GA signal transduction pathways (Lawit et al., 2010).

Hu et al. (2017) found that the maize heterozygotes with higher tolerant to GA inhibitors had higher PH; however, the relationship between GA inhibitor and PH was not significant in inbred lines. Hu’s results indicated that heterozygosity might increase GA levels in maize. A recent study (Xiao et al., 2021) tried to explain why heterozygotes had higher PH compared to the homozygotes. By using a CUBIC synthetic population, two genes, *BR2* on Chromosome 1 and *ubi3* on Chromosome 6, related to PH were detected (Xiao et al., 2021). The recessive *BR2-aa* genotype might repress *ubi3-AA* in a homozygous maternal line; however, in *F*_1_ hybrids, the recessive *BR2-aa* was complemented by a dominant *BR2-T* from the paternal genome, and the heterozygous *BR2-Ta* relieved the suppression of *ubi3*, which causes the high PH in offspring (Xiao et al., 2021). Our study and previous research had suggested that the *Qtl-chr1-EP* might regulate GA in maize and it might even possibly regulate *BR2-aa* since it is located in the promoter region of the *Zm00001d031938*. Further study on this new gene of *Qtl-chr1-EP* might give us a tool to manipulate PH and EH in maize breeding programs aimed at improving planting density in the field.

### Genetic effect of PH and EH on triangle heterotic pattern

The phenotypic data collected here showed large variation in PH and EH across the population in this study, and the two traits were found to be highly correlated. Their ratio (EP) determined the position of the ear on the plant, which is closely related to lodging resistance. Significant genotypic differences were detected for PH and EH, and a wide phenotypic variation was observed across parents and each RIL. Plant height and EH were regulated by many genes, and the interactions between the genes affected the phenotypes of inbred lines and their offspring. In this study, the PH and EH of TRL02 were the lowest among all female parents (Table 2). However, when TRL02 was crossed to Ye107, which is short in PH and EH, the mean PH and EH of their offspring were not the lowest in all seven RIL populations. The result revealed that the heterotic effect of PH and EH between TRL02 and Ye107 played an important role in lowering PH and EH in a hybrid. Actually, from TRL02 × Ye107, a hybrid Yunrui88 had been developed under “Non-Reid × Reid” heterotic pattern (Figure 1). The PH of Yunrui88 is about 250 cm and EH is about 105 cm. The erect plant architecture with compatible PH and EH is a key reason for many farmers accepting Yunrui88 since it can be planted with 30% higher density compared to traditional varieties. The high density with Yunrui88 enhanced maize yield to 17 tons per hectare, which broke the yield record in low-latitude and high-elevation regions in 2010. Yunrui88 has been successfully popularized since 2008 in Southwest China; its accumulated planting area reached 700 thousand hectares, with a newly added output value of 7 billion USD. The success of Yunrui88 affirmed that low PH and EP are very useful traits for high-yielding maize hybrids. The *Qtl-chr1-EP* identified in this study could be a useful tool for balancing EP and grain yield in future maize breeding programs.

## Data availability statement

The datasets presented in this study can be found in online repositories. The names of the repository/repositories and accession number(s) can be found at: https://ngdc.cncb.ac.cn/gsa/, PRJCA009949.

## Author contributions

XF contributed to the conception of the study. XY, YB, and FJ performed the experiment. XF, XY, YB, and FJ contributed significantly to the analysis and manuscript preparation. XY, YB, FJ, and RG performed the data analyses and wrote the manuscript. YZ, JF, and MK helped to perform the analysis with constructive discussions. All authors contributed to the article and approved the submitted version.

## Funding

The study was supported by the National Natural Science Foundation of China (grant no. 31961143014), the Provincial Academician (Expert) Workstation Project of Yunnan (202005AF150026), and the Provincial Major Science and Technology Projects of Yunnan (202102AE090023).

## Conflict of interest

The authors declare that the research was conducted in the absence of any commercial or financial relationships that could be construed as a potential conflict of interest.

## Publisher’s note

All claims expressed in this article are solely those of the authors and do not necessarily represent those of their affiliated organizations, or those of the publisher, the editors and the reviewers. Any product that may be evaluated in this article, or claim that may be made by its manufacturer, is not guaranteed or endorsed by the publisher.

## Footnotes

^1^ https://www.cog-genomics.org/plink/1.9/
