# Supplementary material for: Fine mapping of candidate quantitative trait loci for plant and ear height in a maize nested-association mapping population
Source: Front Plant Sci. 2022 Aug 4;13:963985. doi: 10.3389/fpls.2022.963985 (PMC9386523; doi:10.3389/fpls.2022.963985)
Supplement: Supplementary file 1 [file Data_Sheet_1.pdf]

Supplementary Material

1 Supplementary Tables

Supplemental Table 1. Annotation of SNP variants

	Category	Number of SNPs
Exonic	Stop gain	103
	Stop loss	22
	Synonymous	2994
	Non-synonymous	3301
	unknowns	0
	Upstream	4320
	missense	0
	Intronic	15002
	Splicing	52
	UTR3	3149
	UTR5	1897
	UTR5;UTR3	519
	Downstream	5071
	upstream/downstream	280
	Intergenic	227984
	ts	195082
	tv	69612
	ts/tv	2.802
	Total	264694

Supplemental Table 2. GWAS signals of maize NAM population

Pheno	Chr	Position	P.value(-log10)
EH.BLUP	1	207482464	4.559666985
EH.BLUP	1	207482494	5.797210256
EH.BLUP	1	207482507	5.218006303
EH.BLUP	1	207639576	4.563364635
EH.BLUP	1	208281050	4.706687501
EH.BLUP	1	208673533	5.518095679
EH.BLUP	1	208673540	5.46060648
EH.BLUP	1	208673575	5.752325788
EH.BLUP	1	210043412	4.695113855
EH.BLUP	1	210665124	5.104639127
EH.BLUP	1	210665164	5.210352333
EH.BLUP	1	210665314	4.706926133
EH.BLUP	1	246468926	4.715791382
EH.BLUP	1	246468927	4.98625835
EH.BLUP	1	246468944	4.833293377
EH.BLUP	1	246468972	4.864847079
EH.BLUP	1	246468978	4.708992272
EH.BLUP	1	246468988	5.147617823
EH.BLUP	4	35832257	4.636371329
EH.BLUP	6	46175100	4.624864208
EH.BLUP	6	86703193	4.568860237
EH.BLUP	6	137707961	5.195433983
EH.BLUP	7	46200395	4.788969604
EH.BLUP	7	64433742	6.301457813
EH.BLUP	8	3337902	4.658492943
EH.BLUP	8	93944747	5.165075381
EH_BS	1	49662643	5.075542147
EH_BS	1	207482464	4.786451331
EH_BS	1	207482494	5.860476027
EH_BS	1	207482507	5.206189179
EH_BS	1	246468926	4.777243562
EH_BS	1	246468927	5.062914847
EH_BS	1	246468944	4.791453147
EH_BS	1	246468972	4.857079521
EH_BS	1	246468978	4.811996013
EH_BS	1	246468988	5.094702945
EH_BS	1	252339252	4.704393816
EH_BS	5	110227265	4.739747748
EH_BS	5	110227371	4.768510612
EH_BS	6	46175100	5.143912644
EH_BS	6	46175172	4.97208901

EH_BS	6	76107590	4.784365628
EH_BS	6	86703175	5.49165611
EH_BS	6	86703193	5.178280201
EH_BS	6	137707961	5.474490095
EH_BS	7	64433742	5.052713166
EH_BS	7	83093842	4.803447028
EH_BS	7	120158598	4.862313275
EH_BS	7	120158619	4.806734801
EH_BS	8	127325009	5.03853836
EH_DH	1	208281050	5.076780339
EH_DH	1	208673533	4.789269182
EH_DH	1	208673540	4.668388103
EH_DH	1	208673575	4.812212124
EH_DH	1	210043412	6.317816719
EH_DH	1	210043418	6.17055433
EH_DH	1	210043476	6.01295934
EH_DH	1	210215708	4.736904642
EH_DH	1	254684146	4.828381748
EH_DH	5	131972580	4.839710073
EH_DH	5	131972649	4.904533447
EH_DH	5	131972668	4.915761239
EH_DH	5	136999867	4.64888873
EH_DH	7	64433742	4.674202817
EH_DH	9	58065933	4.866435343
EH_DH	9	58065944	4.563579475
EH_DH	9	58065953	4.632234849
EH_DH	9	58065985	5.214018404
PH.BLUP	1	206008766	4.679253429
PH.BLUP	1	206090508	4.647606609
PH.BLUP	1	207482464	4.750209017
PH.BLUP	1	207482494	5.961917617
PH.BLUP	1	207482507	5.327623384
PH.BLUP	1	208281050	4.805978887
PH.BLUP	1	208669508	4.680874345
PH.BLUP	1	208673533	5.27808254
PH.BLUP	1	208673540	5.257061444
PH.BLUP	1	208673575	5.45407778
PH.BLUP	1	208751861	4.979633207
PH.BLUP	1	210043412	6.528744782
PH.BLUP	1	210043418	5.861725891
PH.BLUP	1	210043476	5.736083903
PH.BLUP	1	210665124	4.798270773
PH.BLUP	1	210665164	4.962716575
PH.BLUP	1	210665314	4.742534333

PH.BLUP	1	246468926	4.804321874
PH.BLUP	1	246468927	4.922530585
PH.BLUP	1	246468944	4.959296834
PH.BLUP	1	246468972	4.918489794
PH.BLUP	1	246468978	4.552052814
PH.BLUP	1	246468988	5.088655092
PH.BLUP	1	246469161	4.583528129
PH.BLUP	1	252312837	4.909935564
PH.BLUP	2	20879902	4.5772216
PH.BLUP	2	20879913	4.680561961
PH.BLUP	2	20879938	4.567760419
PH.BLUP	2	21796615	4.553785731
PH.BLUP	2	140046410	5.027373936
PH.BLUP	2	140046422	4.952913624
PH.BLUP	2	160038044	5.601770121
PH.BLUP	2	160038076	4.892507707
PH.BLUP	2	160038141	4.574196059
PH.BLUP	5	89816073	4.973597927
PH.BLUP	5	89816174	5.809788963
PH.BLUP	5	89816181	5.740671731
PH.BLUP	5	89816200	4.959881882
PH.BLUP	6	46175100	5.19181768
PH.BLUP	6	46175172	4.984942343
PH.BLUP	6	86703175	4.616307033
PH.BLUP	6	137707961	6.800282198
PH.BLUP	10	149356827	4.783845434
PH_BS	1	207482494	5.391272139
PH_BS	1	207482507	4.665793113
PH_BS	1	210665124	4.732821718
PH_BS	1	210665164	4.865800339
PH_BS	1	210665314	4.680994772
PH_BS	2	140046410	5.926809304
PH_BS	2	140046422	5.938649034
PH_BS	2	160038044	4.843063475
PH_BS	2	160038076	4.725127019
PH_BS	6	137707961	5.278786306
PH_BS	8	42564592	4.712113796
PH_BS	8	100232891	4.64963315
PH_DH	1	202365107	5.359216808
PH_DH	1	202365114	5.290072899
PH_DH	1	202365143	4.643564669
PH_DH	1	202365151	4.585620647
PH_DH	1	208673533	5.126163152

PH_DH	1	208673540	5.120724444
PH_DH	1	208673575	5.26444023
PH_DH	1	210043412	7.165439073
PH_DH	1	210043418	6.644315083
PH_DH	1	210043476	6.469602141
PH_DH	1	210215708	5.004366647
PH_DH	2	191362024	4.731267351
PH_DH	4	231434376	5.290675004
PH_DH	4	231434390	5.442181274
PH_DH	4	231434419	5.046633196
PH_DH	5	89816073	5.63226854
PH_DH	5	89816174	6.256803398
PH_DH	5	89816181	6.188072902
PH_DH	5	89816200	4.868024453
PH_DH	5	186483923	4.794282751
PH_DH	6	23027769	4.621294807
PH_DH	6	46175100	4.986236425
PH_DH	6	46175172	4.832533293
PH_DH	6	137707961	7.378415525
EP.BLUP	8	127325009	4.642257171
EP.BLUP	9	43004112	4.589571921
EP.BLUP	10	35248167	5.637471948
EP.BLUP	10	62334395	4.911696579
EP_BS	2	236959420	4.985024408
EP_BS	5	36920481	5.219970656
EP_BS	5	36920490	5.310017786
EP_BS	5	36920496	5.158457673
EP_BS	5	36920505	5.017365633
EP_BS	6	78259284	4.65259407
EP_BS	8	105933692	4.559904491
EP_BS	8	127325009	6.118588222
EP_BS	8	132348643	4.622164476
EP_BS	8	134406114	4.783193359
EP_BS	8	134725969	4.810278726
EP_BS	9	114021372	5.479541513
EP_BS	9	114021456	5.492588398
EP_DH	1	21656844	4.893110361
EP_DH	1	157840817	4.561513134
EP_DH	1	157840853	4.724947203
EP_DH	9	43004112	4.958922916
EP_DH	10	35248167	4.633759269
EP_DH	10	58353427	4.69252945

Supplemental Table 3. Annotation of candidate genes associated by GWAS for PH and EH phenotypes

Pheno	chr	start	end	peakPOS	ref	alt	peak_value	transcript_ID
EH.BLUP	1	207462464	207502507	207482494	T	C	5.7972103	Zm00001d031938
EH.BLUP	1	207619576	207659576	207639576	C	T	4.5633646	Zm00001d031942
EH.BLUP	1	208261050	208301050	208281050	T	G	4.7066875	NA
EH.BLUP	1	208653533	208693575	208673575	C	A	5.7523258	Zm00001d031985
EH.BLUP	1	210023412	210063412	210043412	G	A	4.6951139	NA
EH.BLUP	1	210645124	210685314	210665164	A	G	5.2103523	NA
EH.BLUP	1	246448926	246488988	246468988	G	T	5.1476178	NA
EH.BLUP	4	35812257	35852257	35832257	T	C	4.6363713	NA
EH.BLUP	6	46155100	46195100	46175100	T	C	4.6248642	Zm00001d035762
EH.BLUP	6	86683193	86723193	86703193	G	A	4.5688602	NA
EH.BLUP	6	137687961	137727961	137707961	G	A	5.195434	Zm00001d037785
EH.BLUP	7	46180395	46220395	46200395	C	T	4.7889696	NA
EH.BLUP	7	64413742	64453742	64433742	T	C	6.3014578	NA
EH.BLUP	8	3317902	3357902	3337902	G	A	4.6584929	Zm00001d008258
EH.BLUP	8	3317902	3357902	3337902	G	A	4.6584929	Zm00001d008259
EH.BLUP	8	93924747	93964747	93944747	G	T	5.1650754	NA
EH_BS	1	49642643	49682643	49662643	C	T	5.0755421	Zm00001d028887
EH_BS	1	207462464	207502507	207482494	T	C	5.860476	Zm00001d031938
EH_BS	1	246448926	246488988	246468988	G	T	5.0947029	NA
EH_BS	1	252319252	252359252	252339252	C	T	4.7043938	Zm00001d033154
EH_BS	1	252319252	252359252	252339252	C	T	4.7043938	Zm00001d033155
EH_BS	1	252319252	252359252	252339252	C	T	4.7043938	Zm00001d033156
EH_BS	5	110207265	110247371	110227371	G	A	4.7685106	NA
EH_BS	6	46155100	46195172	46175100	T	C	5.1439126	Zm00001d035762

EH_BS	6	76087590	76127590	76107590	C	G	4.7843656	NA
EH_BS	6	86683175	86723193	86703175	C	A	5.4916561	NA
EH_BS	6	137687961	137727961	137707961	G	A	5.4744901	Zm00001d037785
EH_BS	7	64413742	64453742	64433742	T	C	5.0527132	NA
EH_BS	7	83073842	83113842	83093842	A	G	4.803447	NA
EH_BS	7	120138598	120178619	120158598	A	G	4.8623133	NA
EH_BS	8	127305009	127345009	127325009	C	T	5.0385384	Zm00001d010762
EH_BS	8	127305009	127345009	127325009	C	T	5.0385384	Zm00001d010763
EH_BS	8	127305009	127345009	127325009	C	T	5.0385384	Zm00001d010764
EH_DH	1	208261050	208301050	208281050	T	G	5.0767803	NA
EH_DH	1	208653533	208693575	208673575	C	A	4.8122121	Zm00001d031985
EH_DH	1	210023412	210063476	210043412	G	A	6.3178167	NA
EH_DH	1	210195708	210235708	210215708	G	A	4.7369046	Zm00001d032040
EH_DH	1	254664146	254704146	254684146	C	T	4.8283817	Zm00001d033218
EH_DH	5	131952580	131992668	131972668	G	A	4.9157612	NA
EH_DH	5	136979867	137019867	136999867	T	C	4.6488887	NA
EH_DH	7	64413742	64453742	64433742	T	C	4.6742028	NA
EH_DH	9	58045933	58085985	58065985	C	T	5.2140184	NA
PH.BLUP	1	205988766	206028766	206008766	C	T	4.6792534	Zm00001d031899
PH.BLUP	1	206070508	206110508	206090508	A	G	4.6476066	Zm00001d031901
PH.BLUP	1	207462464	207502507	207482494	T	C	5.9619176	Zm00001d031938
PH.BLUP	1	208261050	208301050	208281050	T	G	4.8059789	NA
PH.BLUP	1	208649508	208693575	208673575	C	A	5.4540778	Zm00001d031985
PH.BLUP	1	208731861	208771861	208751861	T	C	4.9796332	Zm00001d031986
PH.BLUP	1	208731861	208771861	208751861	T	C	4.9796332	Zm00001d031987
PH.BLUP	1	210023412	210063476	210043412	G	A	6.5287448	NA
PH.BLUP	1	210645124	210685314	210665164	A	G	4.9627166	NA
PH.BLUP	1	246448926	246489161	246468988	G	T	5.0886551	NA
PH.BLUP	1	252292837	252332837	252312837	T	C	4.9099356	NA
PH.BLUP	2	20859902	20899938	20879913	A	G	4.680562	NA
PH.BLUP	2	21776615	21816615	21796615	G	A	4.5537857	Zm00001d002757
PH.BLUP	2	21776615	21816615	21796615	G	A	4.5537857	Zm00001d002758

PH.BLUP	2	140026410	140066422	140046410	A	G	5.0273739	Zm00001d004800
PH.BLUP	2	160018044	160058141	160038044	C	A	5.6017701	NA
PH.BLUP	5	89796073	89836200	89816174	G	A	5.809789	Zm00001d015433
PH.BLUP	6	46155100	46195172	46175100	T	C	5.1918177	Zm00001d035762
PH.BLUP	6	86683175	86723175	86703175	C	A	4.616307	NA
PH.BLUP	6	137687961	137727961	137707961	G	A	6.8002822	Zm00001d037785
PH.BLUP	10	149336827	149376827	149356827	C	A	4.7838454	Zm00001d026645
PH.BLUP	10	149336827	149376827	149356827	C	A	4.7838454	Zm00001d026647
PH.BLUP	10	149336827	149376827	149356827	C	A	4.7838454	Zm00001d026648
PH_BS	1	207462494	207502507	207482494	T	C	5.3912721	Zm00001d031938
PH_BS	1	210645124	210685314	210665164	A	G	4.8658003	NA
PH_BS	2	140026410	140066422	140046422	G	A	5.938649	Zm00001d004800
PH_BS	2	160018044	160058076	160038044	C	A	4.8430635	NA
PH_BS	6	137687961	137727961	137707961	G	A	5.2787863	Zm00001d037785
PH_BS	8	42544592	42584592	42564592	C	T	4.7121138	Zm00001d009205
PH_BS	8	100212891	100252891	100232891	C	T	4.6496331	NA
PH_DH	1	202345107	202385151	202365107	G	A	5.3592168	Zm00001d031806
PH_DH	1	208653533	208693575	208673575	C	A	5.2644402	Zm00001d031985
PH_DH	1	210023412	210063476	210043412	G	A	7.1654391	NA
PH_DH	1	210195708	210235708	210215708	G	A	5.0043666	Zm00001d032040
PH_DH	2	191342024	191382024	191362024	C	T	4.7312674	Zm00001d005873
PH_DH	4	231414376	231454419	231434390	C	T	5.4421813	Zm00001d053438
PH_DH	5	89796073	89836200	89816174	G	A	6.2568034	Zm00001d015433
PH_DH	5	186463923	186503923	186483923	G	A	4.7942828	Zm00001d017130
PH_DH	5	186463923	186503923	186483923	G	A	4.7942828	Zm00001d017131
PH_DH	6	23007769	23047769	23027769	C	T	4.6212948	NA
PH_DH	6	46155100	46195172	46175100	T	C	4.9862364	Zm00001d035762
PH_DH	6	137687961	137727961	137707961	G	A	7.3784155	Zm00001d037785
EP.BLUP	8	127305009	127345009	127325009	C	T	4.6422572	Zm00001d010762
EP.BLUP	8	127305009	127345009	127325009	C	T	4.6422572	Zm00001d010763
EP.BLUP	8	127305009	127345009	127325009	C	T	4.6422572	Zm00001d010764

EP.BLUP	9	42984112	43024112	43004112	G	C	4.5895719	NA
EP.BLUP	10	35228167	35268167	35248167	T	G	5.6374719	NA
EP.BLUP	10	62314395	62354395	62334395	G	A	4.9116966	Zm00001d024292
EP.BLUP	10	62314395	62354395	62334395	G	A	4.9116966	Zm00001d024293
EP_BS	2	236939420	236979420	236959420	A	G	4.9850244	Zm00001d007652
EP_BS	5	36900481	36940505	36920490	G	A	5.3100178	NA
EP_BS	6	78239284	78279284	78259284	A	G	4.6525941	Zm00001d036230
EP_BS	8	105913692	105953692	105933692	C	T	4.5599045	Zm00001d010248
EP_BS	8	127305009	127345009	127325009	C	T	6.1185882	Zm00001d010762
EP_BS	8	127305009	127345009	127325009	C	T	6.1185882	Zm00001d010763
EP_BS	8	127305009	127345009	127325009	C	T	6.1185882	Zm00001d010764
EP_BS	8	132328643	132368643	132348643	T	C	4.6221645	Zm00001d010892
EP_BS	8	134386114	134426114	134406114	G	A	4.7831934	Zm00001d010942
EP_BS	8	134386114	134426114	134406114	G	A	4.7831934	Zm00001d010943
EP_BS	8	134386114	134426114	134406114	G	A	4.7831934	Zm00001d010944
EP_BS	8	134705969	134745969	134725969	C	T	4.8102787	NA
EP_BS	9	114001372	114041456	114021456	T	G	5.4925884	NA
EP_DH	1	21636844	21676844	21656844	A	G	4.8931104	NA
EP_DH	1	157820817	157860853	157840853	G	A	4.7249472	Zm00001d030745
EP_DH	9	42984112	43024112	43004112	G	C	4.9589229	NA
EP_DH	10	35228167	35268167	35248167	T	G	4.6337593	NA
EP_DH	10	58333427	58373427	58353427	A	G	4.6925294	Zm00001d024227
EP_DH	10	58333427	58373427	58353427	A	G	4.6925294	Zm00001d024228

Supplemental Table 4. QTLs located in three RIL populations

Pop.	Trait	QTL	Chr	Position(cM)	LOD	Additive effect	Dominant effect	R2
CML312	PH	QTL_{PH}	4	352.81	2.614549	11.5118	0	0.076677
CML312	EH	QTL_{EH}	1	417.51	2.67101	-4.9057	0	0.071404
CML312	EP	QTL_{EP}	1	417.61	2.579805	-0.0164	0	0.067695
D39	PH	$QTL1_{PH}$	1	62.71	4.060803	-8.141	0	0.099773
D39	PH	$QTL2_{PH}$	1	72.91	2.838219	-6.4119	0	0.058696
D39	PH	$QTL3_{PH}$	1	258.81	3.485342	-8.0321	0	0.071515
D39	EH	$QTL1_{EH}$	7	166.71	5.150923	8.3333	0	0.109641
D39	EH	$QTL2_{EH}$	7	186.71	2.638436	-5.7303	0	0.056512
D39	EP	$QTL1_{EP}$	1	147.71	3.93051	-0.0331	0	0.087208
D39	EP	$QTL2_{EP}$	3	422.31	2.736156	-0.0296	0	0.141603
D39	EP	$QTL3_{EP}$	6	167.21	3.774159	0.0197	0	0.091534
D39	EP	$QTL4_{EP}$	6	189.81	3.509229	0.0203	0	0.076262
Y32	EH	$QTL1_{EH}$	2	39.41	4.681868	-8.5447	0	0.130797
Y32	EH	$QTL2_{EH}$	4	105.31	3.124864	5.7225	0	0.131509
Y32	EH	$QTL3_{EH}$	4	111.31	3.77633	6.0507	0	0.149375
Y32	EP	$QTL1_{EP}$	1	129.81	2.690554	-0.0173	0	0.062739
Y32	EP	$QTL2_{EP}$	1	235.11	2.566775	0.0182	0	0.075468
Y32	EP	$QTL3_{EP}$	3	284.51	2.527687	-0.0158	0	0.059941
Y32	EP	$QTL4_{EP}$	6	191.71	3.172638	-0.0223	0	0.091879

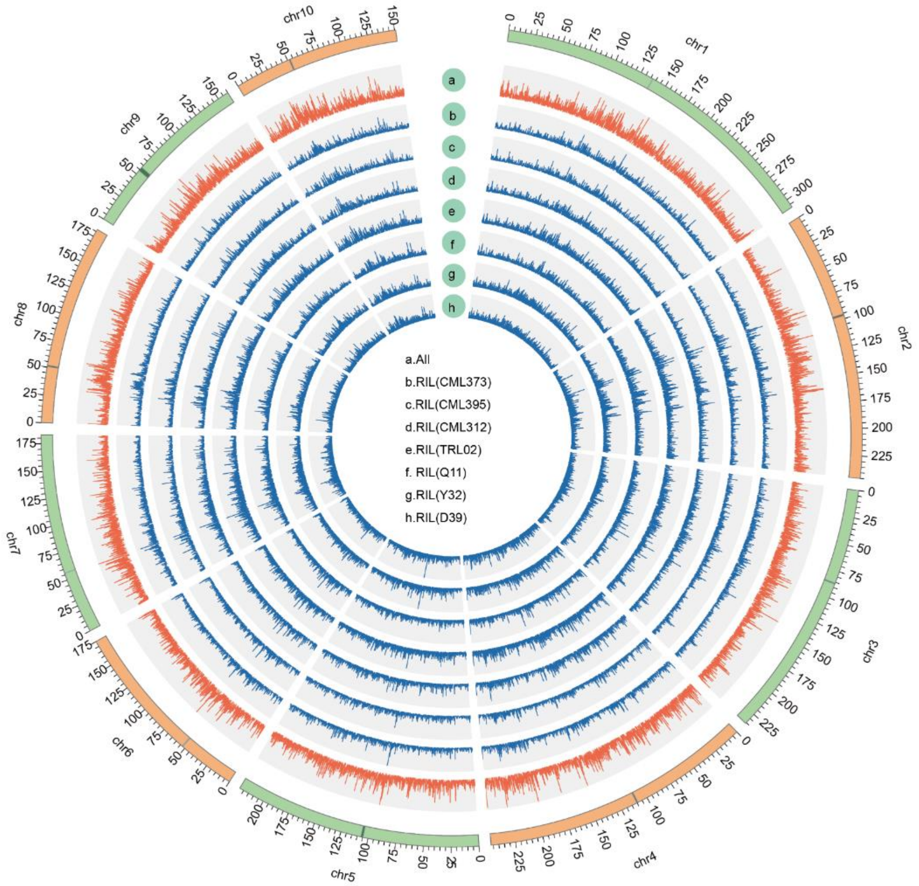

Supplemental Figure 1. Distribution of SNP on 10 chromosomes in seven RIL populations

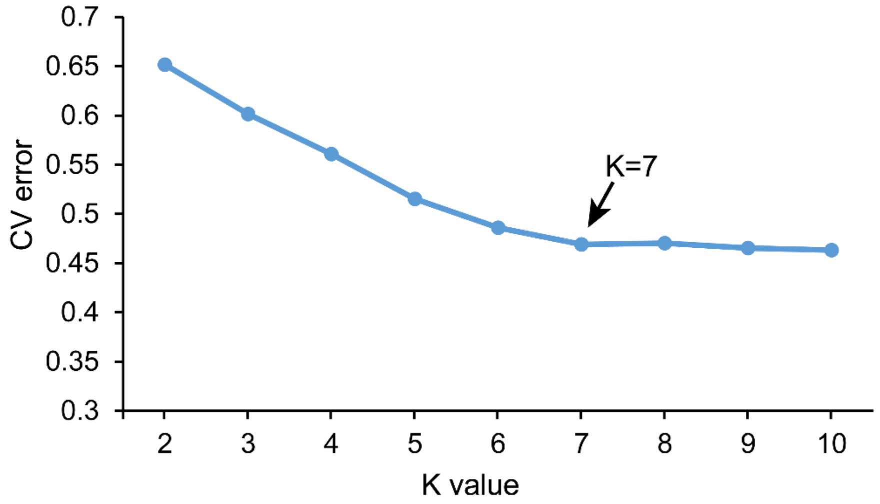

Supplemental Figure 2. The CV error plotted for different values of K showed the lowest error rate at K=7.

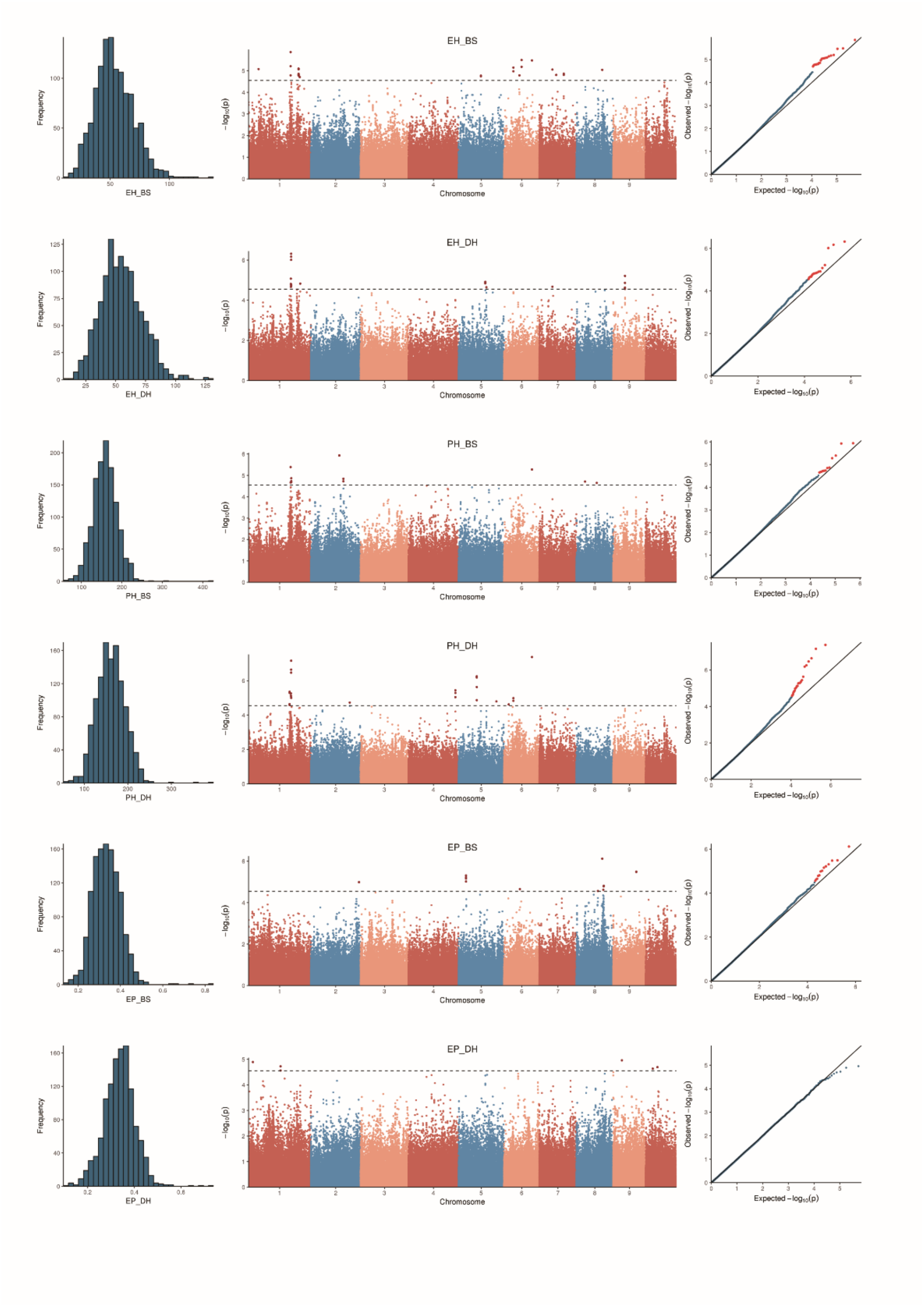

Supplemental Figure 3. Phenotypic distribution, Manhattan and QQ-plot of genome-wide association study for ear height (EH), plant height (PH) and ratio of EH/PH in Baoshan (BS) and Dehong (DH).

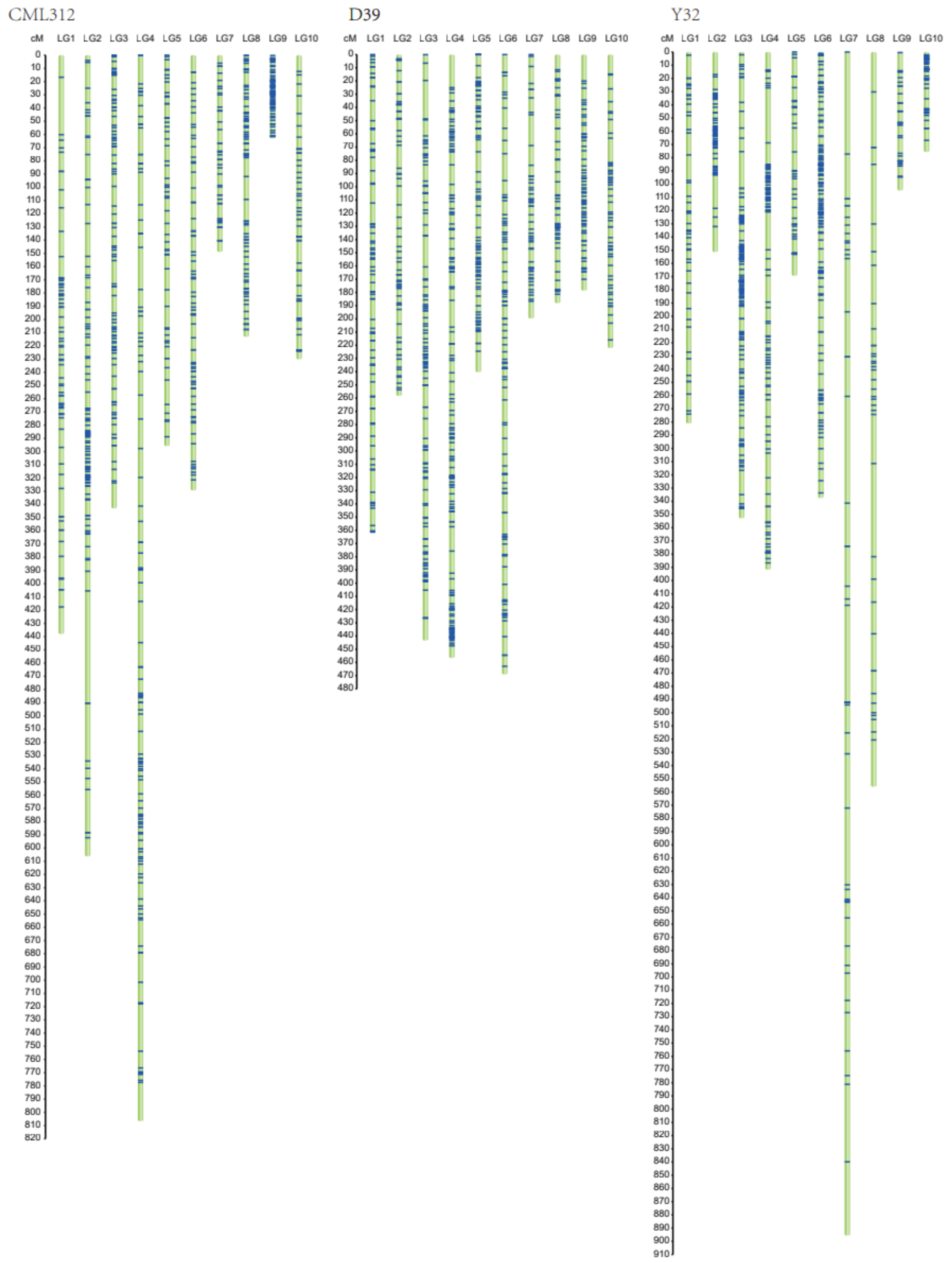

Supplemental Figure 4. Genetic linkage map of CML312, D39 and Y32 RIL population.

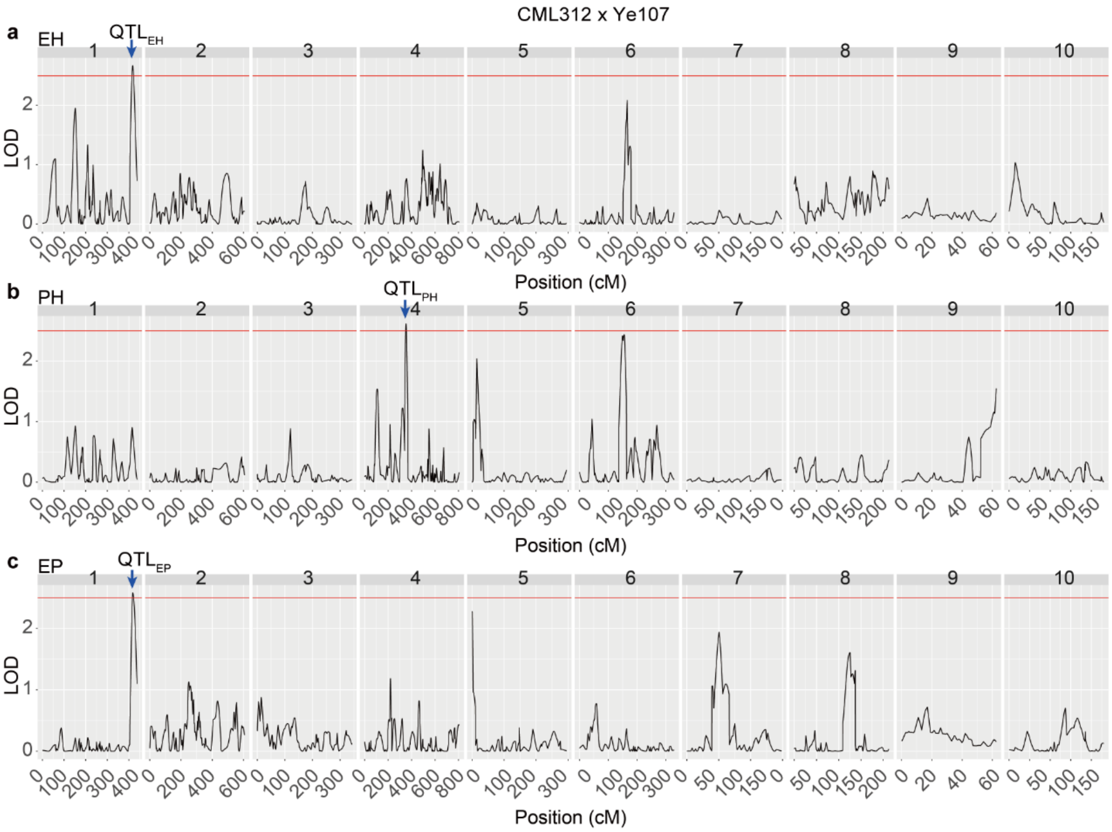

Supplemental Figure 5. QTL map of CML312 RIL population for EH (a), PH (b) and EP (c).

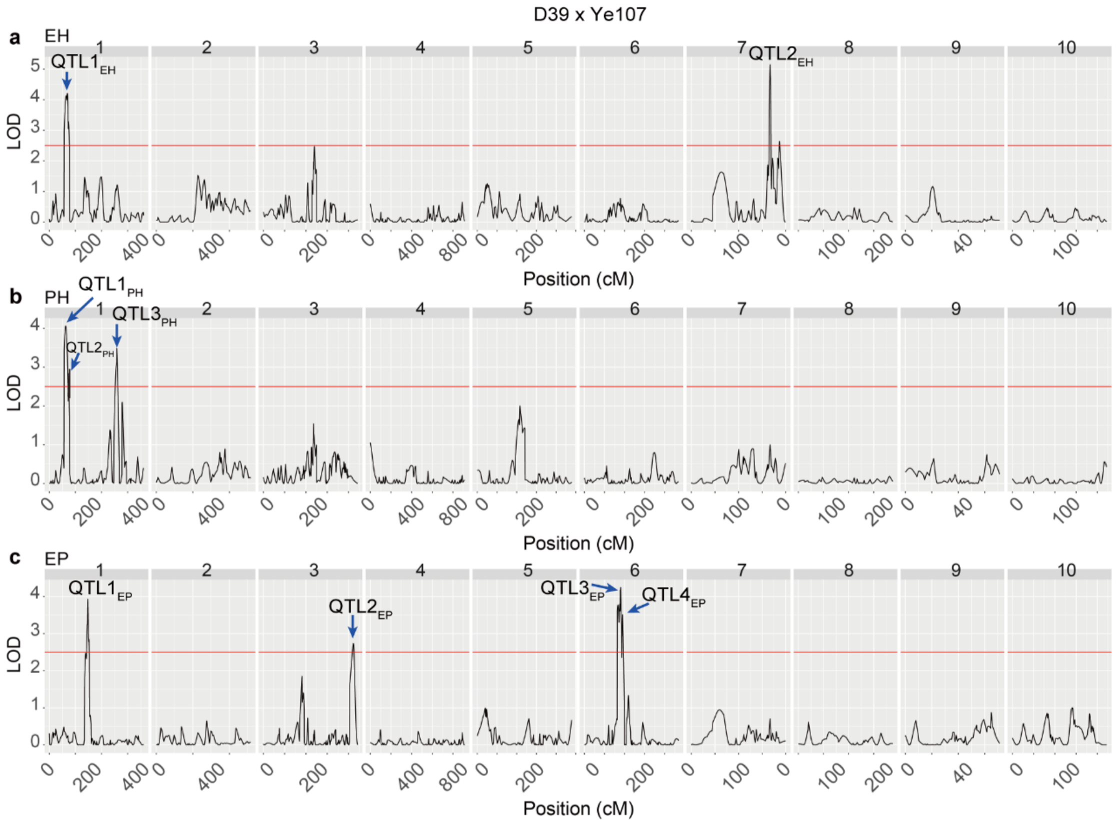

Supplemental Figure 6. QTL map of D39 RIL population for EH (a), PH (b) and EP (c).

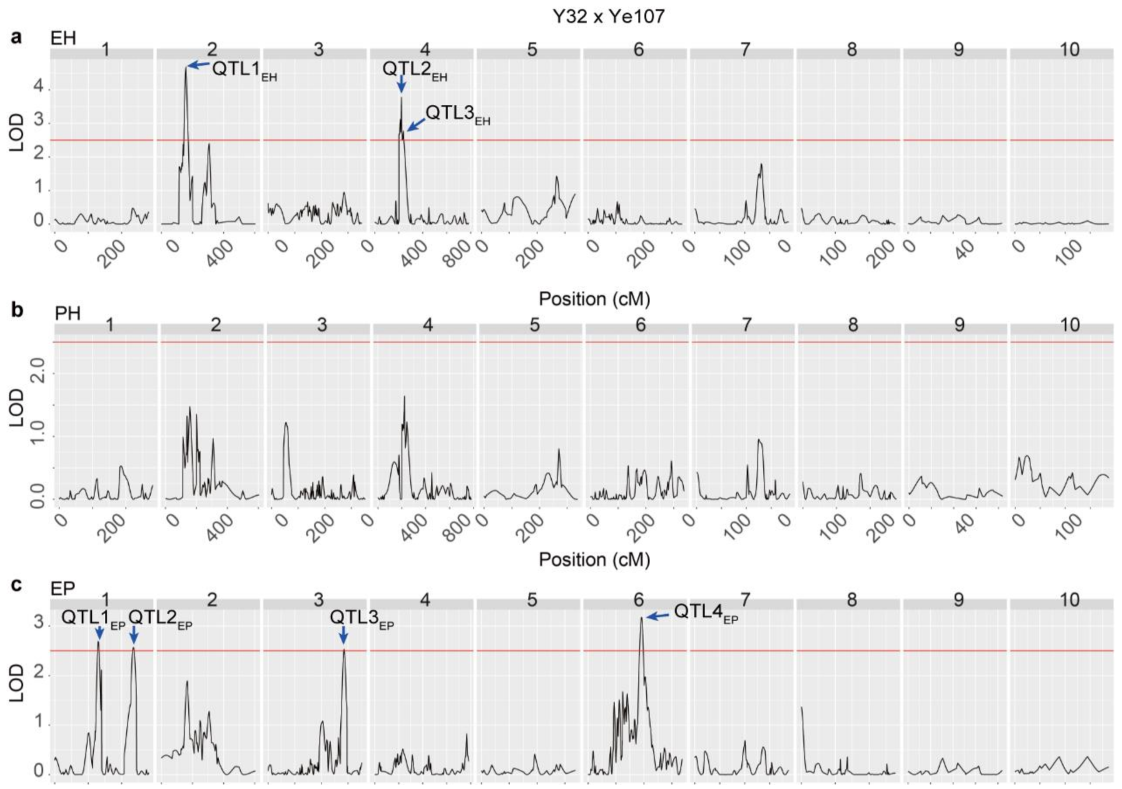

Supplemental Figure 7. QTL map of Y32 RIL population for EH (a), PH (b) and EP (c)
